# Quadrupling the depairing current density in the iron-based superconductor SmFeAsO_1–*x*_H_*x*_

**DOI:** 10.1038/s41563-024-01952-7

**Published:** 2024-07-18

**Authors:** Masashi Miura, Serena Eley, Kazumasa Iida, Kota Hanzawa, Jumpei Matsumoto, Hidenori Hiramatsu, Yuki Ogimoto, Takumi Suzuki, Tomoki Kobayashi, Toshinori Ozaki, Hodaka Kurokawa, Naoto Sekiya, Ryuji Yoshida, Takeharu Kato, Tatsunori Okada, Hiroyuki Okazaki, Tetsuya Yamaki, Jens Hänisch, Satoshi Awaji, Atsutaka Maeda, Boris Maiorov, Hideo Hosono

**Affiliations:** 1 Graduate School of Science and Technology, Seikei University, Tokyo, Japan; 2 National High Magnetic Field Laboratory, Los Alamos National Laboratory, Los Alamos, NM USA; 3 Fusion Oriented REsearch for disruptive Science and Technology (FOREST), Japan Science and Technology Agency (JST), Tokyo, Japan; 4 Department of Electrical & Computer Engineering, University of Washington, Seattle, WA USA; 5 Department of Physics, Colorado School of Mines, Golden, CO USA; 6 College of Industrial Technology, Nihon University, Chiba, Japan; 7 Laboratory for Materials and Structures, Institute of Innovative Research, Tokyo Institute of Technology, Yokohama, Japan; 8 MDX Research Center for Element Strategy, International Research Frontiers Initiative, Tokyo Institute of Technology, Yokohama, Japan; 9 Department of Basic Science, The University of Tokyo, Tokyo, Japan; 10 Kwansei Gakuin University, Sanda, Japan; 11 The Institute of Advanced Sciences, Yokohama National University, Yokohama, Japan; 12 Department of Electrical and Electronic Engineering, University of Yamanashi, Kofu, Japan; 13 Nanostructures Research Laboratory, Japan Fine Ceramics Center, Nagoya, Japan; 14 Institute for Materials Research, Tohoku University, Sendai, Japan; 15 Takasaki Institute for Advanced Quantum Science, National Institutes for Quantum Science and Technology (QST), Takasaki, Japan; 16 Institute for Technical Physics, Karlsruhe Institute of Technology, Eggenstein-Leopoldshafen, Germany; 17 National Institute for Materials Science (NIMS), Tsukuba, Japan

**Keywords:** Superconducting properties and materials, Superconducting properties and materials

## Abstract

Iron-based 1111-type superconductors display high critical temperatures and relatively high critical current densities *J*_c_. The typical approach to increasing *J*_c_ is to introduce defects to control dissipative vortex motion. However, when optimized, this approach is theoretically predicted to be limited to achieving a maximum *J*_c_ of only ∼30% of the depairing current density *J*_d_, which depends on the coherence length and the penetration depth. Here we dramatically boost *J*_c_ in SmFeAsO_1–*x*_H_*x*_ films using a thermodynamic approach aimed at increasing *J*_d_ and incorporating vortex pinning centres. Specifically, we reduce the penetration depth, coherence length and critical field anisotropy by increasing the carrier density through high electron doping using H substitution. Remarkably, the quadrupled *J*_d_ reaches 415 MA cm^–2^, a value comparable to cuprates. Finally, by introducing defects using proton irradiation, we obtain high *J*_c_ values in fields up to 25 T. We apply this method to other iron-based superconductors and achieve a similar enhancement of current densities.

## Abstract (web summary)

The authors report very high in-field critical current densities in Fe-based superconductors. They achieve this through doping to increase the carrier density and adding defects to pin vortices.

## Main

Superconductors have attracted technological interest partially due to their extremely high dissipation-free current densities *J*_c_. In these materials, the superconducting current is carried by electron pairs that can be broken apart if the current surpasses the depairing current density *J*_d_, which sets the ultimate limit on the achievable *J*_c_. A considerable challenge at the convergence between fundamental and applied superconductivity research is determining what structural and chemical properties are required to reach this maximum and how *J*_c_ depends on parent or competing phases in the magnetic phase diagram. The critical current can be severely restricted by dissipation triggered by the motion of vortices, magnetic flux lines that penetrate type-II superconductors. Accordingly, most research efforts to increase *J*_c_ have focused on adding material defects, creating spatial inhomogeneities in the free energy and therefore preferential positions for vortices to localize (pin) to reduce their core energies^1–3^. Consequently, the defect landscape defines a vortex pinning potential *U*_act_(*J*, *H*, *T*) that depends on the current density *J*, magnetic field *μ*_0_*H* where *μ*_0_ is the permeability in vacuum and *H* is the magnetic field strength, temperature *T* and disorder.

When optimized, this approach of core pinning alone may achieve a maximum *J*_c_ of only ∼30% of *J*_d_. The argument for this specific limit first considers how, to immobilize vortices, the effective pinning force imparted by the collective action of defects must balance out the force induced by the current. It then finds that the maximum pinning force *f*_p_^core^ imposed by pinning vortex cores (reducing the vortex energy by the condensation energy) is only ∼30% that of the driving force *f*_d_ induced from a current equivalent to *J*_d_, such that *f*_p_^core^/*f*_d_ ≈ 30% (refs. ^4–6^). (As such, a *J*_c_ near *J*_d_ has been achieved only in a nanomesh system precluding vortex formation, but this architecture is not scalable for large-scale applications^7^.) Most materials achieve critical currents far below this ceiling given the challenges associated with optimizing this pinning potential—notably, incorporating defects without straining the interlaying superconducting matrix and inadvertently suppressing *J*_c_ or the critical temperature *T*_c_.

**Fig. 1:**
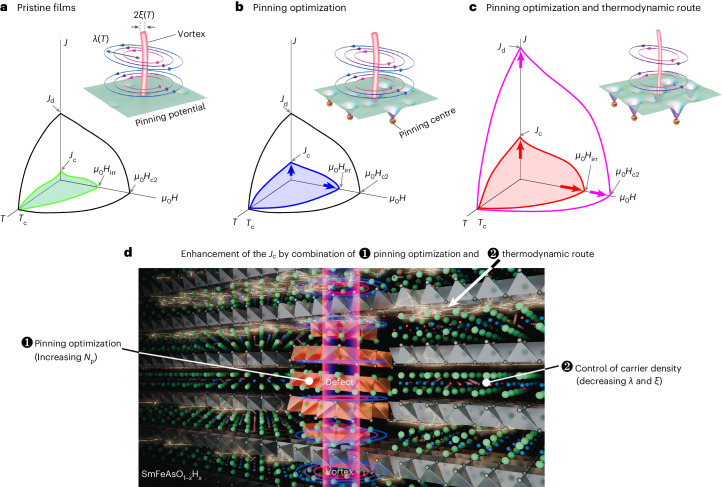
Evolution of the phase diagram of superconductors after pinning optimization and tuning the carrier density. The *J*–*H*–*T* phase diagrams for superconductors. **a**, As-grown films. **b**, Films after incorporating defects to pin vortices (pinning optimization). **c**, Films after a combined method of tuning the carrier density (thermodynamic route) and pinning optimization. Note that vortex pinning also occurs in the as-grown film, based on the intrinsic defect landscape. The insets depict a vortex navigating a pinning landscape in each scenario. **d**, A schematic of the microstructure resulting from the combined approach. *N*_p_, density of pinning centres.

Here we present a somewhat new paradigm for boosting *J*_c_. We propose targeting increasing *J*_d_ to raise the ceiling—denoted the thermodynamic approach—while concurrently engineering the defect landscape to approach this higher upper limit—collectively referred to herein as the combined approach. The comparative *J*–*H*–*T* phase diagrams are shown in Fig. 1. From Fig. 1a, we see that pristine films typically have small spatial variations in the potential energy landscape, leading to easy vortex motion and therefore a low *J*_c_/*J*_d_ ratio. Figure 1b captures how incorporating defects deepens the wells (*U*_act_) that vortices must overcome to move. Here, *U*_act_ depends on the type of pinning, for example, strong pinning, in which a large defect can independently impart sufficient force on a vortex to immobilize it, or weak collective pinning, in which atomic-scale defects act collectively to immobilize segments of a vortex^8,9^. Accordingly, the *J*_c_/*J*_d_ ratio increases, reaching a maximum at ∼30%—a value achieved in few studies—balanced with the challenge of avoiding degradation in *T*_c_. Lastly, our combined approach is illustrated in Fig. 1c,d. The energy wells deepen not only from defects, but also from fundamentally changing the vortex structure, affecting the condensation energy. By increasing *J*_d_, higher *J*_c_ values are achieved, even if the *J*_c_/*J*_d_ ratio is maintained compared to values from defect engineering alone.

Regarding how to tune *J*_d_, we turn to Ginzburg–Landau theory^10^, which derives the temperature dependence of the depairing current density as

1 $${J}_{{\rm{d}}}(T\;)={\varPhi }_{0}/\left[3\sqrt{3}\uppi {{\mu }_{0}{\rm{\xi}}\left({{T}}\right)(\lambda \left(T\right))}^{2}\right] \propto {n}_{{\rm{s}}}(T\;),$$

where *Φ*_0_ is the flux quantum, *μ*_0_ is the permeability in vacuum, *ξ* is the coherence length, *λ* is the penetration depth and *n*_s_ is the superfluid density. The vortex structure depends on two parameters: the vortex core diameter is ∼2*ξ*(*T*) and it is surrounded by supercurrents of radius up to *λ*(*T*). From equation (1), we see that a dramatic reduction in the penetration depth would induce a concomitantly substantial increase in *J*_d_. The penetration depth depends inversely on the superfluid density *n*_s_ as $\lambda \propto \sqrt{{m}^{* }/{n}_{{\mathrm{s}}}}$, where m* is the effective mass. Hence, we can tune *λ* by controlling the carrier density through doping—the thermodynamic route.

**Fig. 2:**
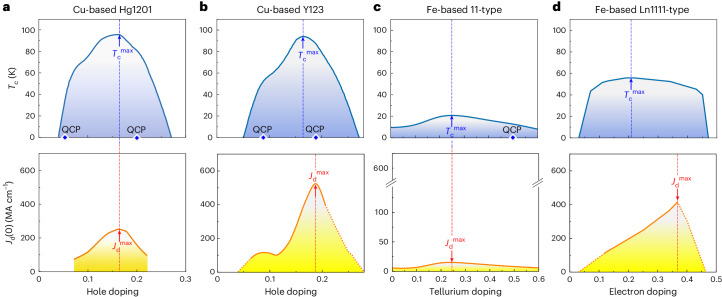
Carrier density dependence of *T*_c_ and *J*_d_. **a**–**d**, Dependence of *T*_c_ (top panel) and *J*_d_ (bottom panel) for Cu-based Hg1201 (**a**; refs. ^12,13^), Cu-based Y123 (**b**; refs. ^3,16,17^), Fe-based 11-type (**c**; refs. ^14,15^) and Fe-based *Ln*1111-type (**d**; refs. ^18,19^) superconductors. The solid curves represent measurements from the citations, whereas the dashed segments in **a**, **b** and **d** (bottom panel) are from our unpublished data. Note that *J*_d_ in **d** is calculated based on *λ* and *ξ*, which are experimentally obtained in this work (Fig. 3). Notice that, for the *Ln*1111-type Fe-based material, though the peaks in *J*_d_ (*J*_d_^max^) and *T*_c_ (*T*_c_^max^) do not occur at the same doping level, the broad *T*_c_–doping dome enables sufficiently high doping to reach the *J*_d_ peak with a minimal decrease in *T*_c_ from its maximum. The QCP in the 11-type superconductor has been observed only in a bulk sample^14^.

To identify appropriate candidates to implement this route for optimizing *J*_c_, we consider the *T*_c_–doping phase diagrams, which can host a variety of phases outside of the superconducting phase. From the *T*_c_–doping phase diagrams, illustrated in Fig. 2, we must identify materials in which the predicted increase in *J*_d_ (Supplementary Table 1) is not accompanied by a rapid decrease in *T*_c_. For materials that follow the Uemura plot^11^ (that is, *T*_c_ ∝ 1/*λ*^2^), *T*_c_ and *J*_d_ should apex at the same carrier density because both parameters are proportional to 1/*λ*^2^. We show examples of this in Fig. 2a,c for the moderately anisotropic Cu-based HgBa_2_CuO_4+*δ*_ (Hg1201) superconductor^12,13^ and nearly isotropic Fe-based FeSe_1–*x*_Te_*x*_ (11-type) superconductor^14,15^, respectively. On the other hand, Fig. 2b,d shows example results from a Cu-based YBa_2_Cu_3_O_*y*_ (Y123) sample^3,16,17^ and an Fe-based SmFeAsO_1–*x*_H_*x*_ (a *Ln*1111-type) sample (*Ln* is a lanthanoid element)^18,19^ in which *J*_d_ and *T*_c_ do not peak at the same doping level, but rather *J*_d_ peaks in the highly doped region because of a non-concurrent dip in *λ* and peak in *T*_c_ with carrier density.

From Fig. 2b,d, we identify Y123 and *Ln*1111-type iron-based superconductors as ideal candidates for our combined approach because *T*_c_ remains nearly maximal when *J*_d_ peaks in the high doping regime. Based on this, we have previously demonstrated extremely high *J*_c_ values in (Y_0.77_Gd_0.23_)Ba_2_Cu_3_O_*y*_ films using our proposed approach, by precisely tuning *J*_d_ through controlling the carrier concentration and then incorporating nanoparticles^3^ to pin vortices. We achieved a critical current density reaching ∼32.4% of *J*_d_ at 4.2 K. From Fig. 2b, we now observe that the peak in *J*_d_ coincides with a quantum critical point (QCP)—a sudden change in the *n*_s_ and effective mass m*. This is quite interesting; the presence of QCPs should have a dramatic impact on *J*_d_ and *J*_c_, providing partial motivation for studying the doping dependence of *J*_c_ in multiple categories of superconductors.

In this study we test our combined approach in iron-based superconductors, with a focus on a *Ln*1111-type material. We successfully achieve very high self-field *J*_c_ (*J*_c_^s.f.^) and in-field *J*_c_ values as well as pinning force densities *F*_p_ for SmFeAsO_1–*x*_H_*x*_ films—among the highest reported for iron-based superconductors. Specifically, at 4.2 K, the samples demonstrate a *J*_c_^s.f.^ ≈ 16.11 MA cm^–2^ and in-field *J*_c_(25 T) = 3.01 MA cm^–2^, corresponding to *F*_p_(25 T) ≈ 750 GN m^–3^, with the latter two measured in an out-of-plane field (**H** ∥ *c*) of 25 T. Additionally, we apply a new method of increasing the carrier concentration well into the highly doped regime by doping with hydrogen^20^, beyond what is achievable using the traditional method of doping with fluorine. This enables tuning into a regime in which we calculate a maximum in *J*_d_(*T* = 0) of 415 MA cm^–2^ (Fig. 2d), surpassed only by overdoped Y123 (refs. ^3,21^). We further successfully apply this approach to other iron-based superconductors. In FeSe_1–*x*_Te_*x*_ films, we find that *J*_c_ at 4.2 K increases from 0.29 to 1.43 MA cm^–2^ due to the increase in *J*_d_(0) by a factor of 3.0. Moreover, for BaFe_2_(As_1–*x*_P_*x*_)_2_ films, *J*_c_ at 4.2 K increases from 0.45 to 3.55 MA cm^–2^ due to the increase in *J*_d_(0) by a factor of 5.0.

## H doping and proton irradiation of SmFeAsO films

For this study we first grew 50-nm-thick films of the parent compound SmFeAsO by pulsed laser deposition on MgO substrates and then doped them with hydrogen. To substitute H^−^ for a fraction of the O^2−^ sites, we performed a topotactic chemical reaction using CaH_2_ powder. This enabled us to increase the carrier density into the highly doped regime—with a high H concentration reaching ∼35% of O sites, higher than that achieved with F doping (<20%). Film preparation process details have been published elsewhere^20,22^. Next we analysed the depth dependence of the H concentrations (*x* in Supplementary Fig. 1). Lastly we irradiated some samples to induce artificial nano-defects (Methods for details).

**Fig. 3:**
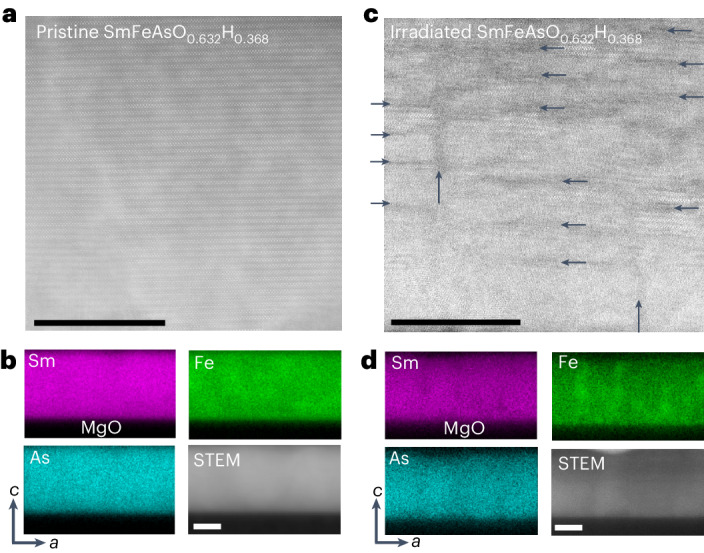
Microstructure of a SmFeAsO_1–*x*_H_*x*_ thin film (*x* = 0.368) grown epitaxially on MgO. **a**, Cross-sectional STEM image of the pristine film. **b**, Elemental maps of the pristine film and low-magnification STEM image. Sm, Fe and As were homogeneously distributed in the matrix; maps collected using EDS. **c**, STEM image of the film after proton irradiation, revealing that irradiation introduced defects oriented along both the *c* axis and *ab* plane. **d**, Elemental maps and low-magnification STEM image of the irradiated film. All scale bars, 20 nm. The vertical and horizontal arrows indicate the nano-sized defects.

Figure 3 compares the microstructure of pristine and irradiated SmAsFeO_0.632_H_0.368_ films, displaying scanning transmission electron microscope (STEM) images and elemental maps (through energy dispersive spectroscopy, EDS) in Fig. 3a,c and Fig. 3b,d, respectively. The STEM image shows that the pristine SmAsFeO_0.632_H_0.368_ film is epitaxial, without discernible defects. In Fig. 3b, the EDS maps of Sm, Fe and As indicate that the film is homogeneous. Figure 3c reveals several nano-sized defects aligned with the *c* axis and *ab* plane, defects that were induced by the proton irradiation process. In Fig. 3d, from the EDS maps we observe some small compositional variations mostly aligned along the *c* axis that appear rich in Fe and poor in Sm and As.

**Fig. 4:**
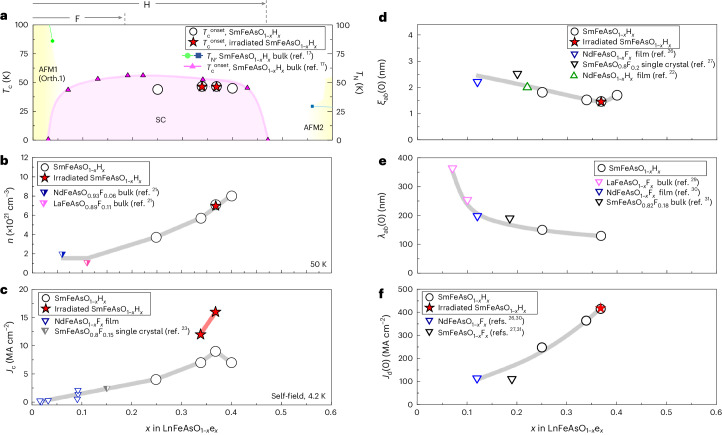
Physical quantities as a function of electron doping (per Fe). **a**, Phase diagram of SmFeAsO. There is a single *T*_c_ dome and two antiferromagnetic phases (AFM1 and AFM2) near *x* < 0.05 and *x* > 0.55 (ref. ^19^). At the top of panel **a**, the arrow labeled F indicates the range of electron doping accessible by doping with fluorine, whereas the arrow labeled H indicates the range accessible by doping with hydrogen. Here, SC refers to the superconducting phase, Orth.1 refers to the structural transition from the tetragonal to the orthorhombic phase, *T*_N_ is the Néel temperature and *T*_c_^onset^ is the onset of the critical temperature. **b**, Carrier density *n* at 50 K for SmFeAsO_1–*x*_H_*x*_ films and *Ln*FeAsO_1–*x*_F_*x*_ (ref. ^23^). **c**, Self-field *J*_c_ at 4.2 K. For a comparison, the data for NdFeAsO_1–*x*_F_*x*_ and SmFeAsO_1–*x*_F_*x*_ (ref. ^25^) are superimposed. **d**, Zero-temperature in-plane coherence length *ξ*_*ab*_(0) evaluated from the slope of the upper critical field for SmFeAsO_1–*x*_H_*x*_ films and *Ln*FeAsO_1–*x*_e_*x*_ (refs. ^24,28,29^). **e**, Zero-temperature in-plane penetration depth *λ*_*ab*_(0) measured by the superconducting resonator method. For a comparison, the data for *Ln*FeAsO_1–*x*_F_*x*_ (refs. ^31–33^) are plotted. **f**, Zero-temperature depairing current density *J*_d_(0) calculated by equation (1); *ξ*_*ab*_(0) and *λ*_*ab*_(0) were taken from **d** and **e**. For the irra**d**iated films, *λ*_*ab*_(0) for the pristine film was used to calculate *J*_d_. In **b–d**, the grey lines are guides to the eye highlighting the trends in each parameter with changes in doping, and the red line highlights the trend for the irradiated sample. Source data

Figure 4a presents the phase diagram for pristine and irradiated films. For reference, the data from SmFeAsO_1–*x*_H_*x*_ bulk samples are also plotted^19^. The *T*_c_ and *J*_c_ values were measured using a four-probe method; data are shown in Supplementary Fig. 2a,b, respectively. The films also demonstrate little sensitivity of *T*_c_ to electron concentration (*x* in Fig. 4; e, electron in this figure), and irradiation reduces *T*_c_ by only 0.4 K for both SmAsFeO_0.661_H_0.339_ and SmAsFeO_0.632_H_0.368_ films. Figure 4b shows the carrier density (*n*) at 50 K as a function of *x* for the pristine and irradiated films obtained from Hall measurements (Supplementary Fig. 3). For a comparison, *n* values for other *Ln*1111-type bulk samples are plotted^23^. The *n* in the SmFeAsO_1–*x*_H_*x*_ films increases with increasing *x*. The *J*_c_^s.f.^ at 4.2 K increases with carrier doping beyond that achieved by F doping, as shown in Fig. 4c. Even though the pristine and irradiated films have almost the same dependence of *T*_c_ and carrier density *n* on carrier doping *x*, the *J*_c_^s.f.^ of the irradiated SmFeAsO_1–*x*_H_*x*_ films is 1.75 times higher than that in the pristine film. This is because *n*, *T*_c_ and *ξ* of the interlaying superconducting matrix, between defect clusters, are unaltered by irradiation. The *J*_c_ values in the irradiated SmAsFeO_0.632_H_0.368_ films (*J*_c_^s.f.^(4.2 K) = 16.11 MA cm^–2^) are the same order of magnitude as those of NdFeAsO_1–*x*_H_*x*_ films^24^ and an irradiated SmFeAsO_1–*x*_F_*x*_ single crystal^25^. Supplementary Table 2 compares *T*_c_, *n* and *J*_c_^s.f.^ values for the pristine versus irradiated films.

## Tuning the depairing current density

In Fig. 4d,e, we show how the in-plane coherence length, *ξ*_*ab*_ and in-plane penetration depth, *λ*_*ab*_ vary with carrier doping. Figure 4d displays *ξ*_*ab*_(*T* = 0) as a function of *x* for our films. The coherence length *ξ*_*ab*_ is typically calculated from measurements of the temperature-dependent upper critical field, *H*_c2_, that is, *μ*_0_*H*_c2_(*T*) = *ϕ*_0_/2π(*ξ*_*ab*_(*T*))^2^, in which *H*_c2_(0) and therefore *ξ*_*ab*_(0) are extracted from an extrapolation of a fit to an appropriate model, given the inability to access fields up to *H*_c2_(0). The Werthamer–Helfand–Hohenberg formula^26^ is most commonly applied, though in its original form it strictly models the case of single-band superconductors. Here we apply the Gurevich two-band model in the clean limit^27^ to fit the experimental *H*_c2_(*T*) data. We find that most of our *H*_c2_(*T*) data fit well to this model, and that the SmAsFeO_0.632_H_0.368_ film exhibits a downward curvature in *H*_c2_(*T*) over a very narrow range of temperatures (within 2% of *T*_c_) that notably deviates from the fit. An in-depth discussion of the fits, speculation on the cause of the downturn in one sample, temperature-dependent resistivity data from which *H*_c2_ is extracted (Supplementary Fig. 4) and the fits (Supplementary Figs. 5 and 6 and Supplementary Table 3) are included in the Supplementary Information. For reference, *ξ*_*ab*_(0) values from the literature for other doped *Ln*1111-type materials are included^24,28,29^. The calculated *ξ*_*ab*_(0) of the SmAsFeO_0.632_H_0.368_ films decreases with increasing *x*, with the pristine and irradiated SmAsFeO_0.632_H_0.368_ achieving the lowest *ξ*_*ab*_(0) value, which is close to the doping level of a possible QCP in NdFeAsO_1–*x*_H_*x*_ (ref. ^30^). Interestingly, local minima of *ξ*_*ab*_(0) at the two QCPs have been reported for Y123 (ref. ^17^). Next, to determine the penetration depth *λ*_*ab*_(*T* = 0), we use the temperature dependence of the resonant frequency of the coplanar waveguide resonators (detailed in Supplementary Fig. 7). As shown in Fig. 4e, we see that *λ*_*ab*_(0) of NdFeAsO_1–*x*_H_*x*_ films decreases with increasing *x*, which is a similar trend to that of other doped *Ln*1111-type materials^31–33^, indicating there are thermodynamic changes occurring with H doping.

Figure 4f displays the calculated *J*_d_(0) within Ginzburg–Landau theory^10^ for other doped *Ln*1111-type materials. The zero-temperature depairing current density *J*_d_(0) for the SmFeAsO_1–*x*_H_*x*_ films increases with *n*, reaching a maximum at a large value of 415 MA cm^–2^. This is among the highest *J*_d_ for any superconductor, surpassed by overdoped Y123 (*J*_d_(0) ≈ 500 MA cm^–2^) and higher than that of Hg1201 (*J*_d_(0) = 280 MA cm^–2^). Supplementary Table 1 shows a comparison of *J*_d_ in multiple superconductors. Because *T*_c_ typically correlates with *J*_d_, it is also remarkable that a material with half the *T*_c_ of Y123 and Hg1201 produces a similar *J*_d_. For our film with maximum doping, the *J*_d_ enhancement is one of the main reasons for the higher *J*_c_, as shown in Fig. 4c.

## High in-field critical current density

**Fig. 5:**
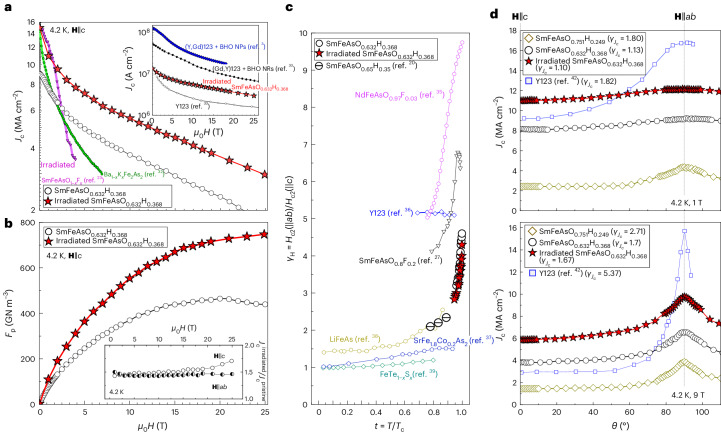
In-field superconducting properties of the pristine and irradiated SmFeAsO_0.632_H_0.368_ samples. **a**, A comparison of the field dependent *J*_c_ for pristine and irradiated SmFeAsO_1–*x*_H_*x*_ films, an irradiated SmFeAsO_1–*x*_F_*x*_ crystal^25^ and a Ba_1–*x*_K_*x*_Fe_2_As_2_ film^34^. The inset in **a** compares results for the irradiated H-doped Sm1111 film to data for an overdoped (Y,Gd)123 conductor containing BaHfO_3_ nanoparticles (BHO NPs)^3^, a Y123 conductor^35^ and (Gd,Y)123 with BaHfO_3_ nanorods (NRs)^35^, showing that *J*_c_ in the SmFeAsO_1–*x*_H_*x*_ is higher than in the Y123 conductor. **b**, *F*_p_ for the pristine SmFeAsO_0.632_H_0.368_ showed a peak of ∼420 GN m^–3^ at around 20 T. After irradiation, the maximum *F*_p_ was 740 GN m^–3^ at around 25 T. The enhancement in *J*_c_ after irradiation as a function of the applied field is shown in the inset in **b**. The ratio of *J*_c_ after irradiation (*J*_c_^irradiated^) to *J*_c_ before (*J*_c_^pristine^) was calculated using the data shown in Supplementary Fig. 8. Notice that the enhancement is maintained up to the highest applied magnetic field for both field orientations. **c**, Anisotropy of *H*_c2_ for the SmFeAsO_0.632_H_0.368_ films as a function of reduced temperature (*t*). Here, *H*_c2_(||*ab*) and *H*_c2_(||*c*) are the upper critical field values when the field is applied parallel to the *ab*-plane and *c*-axis, respectively. The *γ*_*H*_ was slightly reduced by the proton irradiation. For a comparison, the data for NdFeAsO_0.97_F_0.03_ (ref. ^37^), Y123 (ref. ^38^), SrFe_1.8_Co_0.2_As_2_ (ref. ^39^), LiFeAs (ref. ^40^), FeTe_1−*x*_S_*x*_ (ref. ^41^), SmFeAsO_0.65_H_0.35_ (ref. ^22^) and SmFeAsO_0.8_F_0.2_ (ref. ^29^) are included. Compared to NdFeAsO_0.97_F_0.03_ (ref. ^37^) and SmFeAsO_0.8_F_0.2_ (ref. ^29^), SmFeAsO_0.632_H_0.368_ shows lower *γ*_*H*_. **d**, Angular dependence of *J*_c_ at 4.2 K and 1 T (top) as well as 9 T (bottom) for various SmFeAsO_1–*x*_H_*x*_ films. The anisotropy of *J*_c_ is calculated as ${\gamma}_{J_{\mathrm{c}}} = J_{{\mathrm{c}}}^{\;ab}/J_{{\mathrm{c}}}^{\;c}$ for each sample, which is indicated in the legend. Compared to Y123 (ref. ^44^), the ${\gamma}_{J_{\mathrm{c}}}$ of SmFeAsO_0.632_H_0.368_ is very small, especially at 9 T. After irradiation, *J*_c_ increased in SmFeAsO_0.632_H_0.368_ for all field orientations. Source data

The field dependence of *J*_c_ at 4.2 K for **H** ∥ *c* for pristine and irradiated SmAsFeO_0.632_H_0.368_ films is summarized in Fig. 5a, with data for other superconductors included. First, the irradiated SmAsFeO_0.632_H_0.368_ film, irradiated SmFeAsO_1–*x*_F_*x*_ (ref. ^25^) and Ba_1–*x*_K_*x*_Fe_2_As_2_ (ref. ^34^) all have similar *J*_c_^s.f.^ values. However, *J*_c_ for the latter two samples degrades rapidly with increasing field, such that both the pristine and irradiated SmAsFeO_0.632_H_0.368_ films demonstrate the highest *J*_c_ for fields above ∼3.5 T. Second, proton-irradiated SmAsFeO_0.632_H_0.368_ improves the in-field *J*_c_, reaching a high *J*_c_ value for iron-based superconductors^24,25,28,34^. In fact, the in-field *J*_c_ in this film surpasses that of optimally doped Y123 (ref. ^35^), although it remains lower than the current record value, which was achieved in an overdoped Y123 film containing nanoparticles^3^.

The remarkable in-field performance achieved by H doping and subsequently irradiating the SmAsFeO_0.632_H_0.368_ film is highlighted in the pinning force density (*F*_p_) plot in Fig. 5b. For **H** ∥ *c*, *F*_p_ for the pristine SmAsFeO_0.632_H_0.368_ film reaches a maximum at 20 T and then decreases for higher fields. Consistent with the increase in *J*_c_, *F*_p_ for the irradiated SmAsFeO_0.632_H_0.368_ film increased for all applied fields and orientations measured; *F*_p_ at 25 T increased by a factor of 1.7 upon irradiation. The *F*_p_ for the irradiated SmAsFeO_0.632_H_0.368_ film does not saturate up to 25 T, as a result of the improved *J*_c_(*H*) due to enhanced vortex pinning by proton irradiation. Moreover, for both **H** ∥ *c* and **H** ∥ *ab*, the irradiated SmAsFeO_0.632_H_0.368_ film has a roughly 1.5 times higher *J*_c_ compared to that in pristine film at all fields up to 25 T, as seen in the Fig. 5b inset.

## Reduced upper critical field anisotropy

For many materials, *F*_p_ peaks and then dramatically decreases with increasing magnetic field. However, our irradiated SmAsFeO_0.632_H_0.368_ film maintains a relatively high *F*_p_ up to high magnetic fields, which is promising for high-field applications. Moreover, our samples have two additional desirable characteristics for applications: relatively low upper critical field anisotropy and consequently a *J*_c_ that is insensitive to field orientation. Specifically, in Fig. 5c, we show the *H*_c2_ anisotropy *γ*_*H*_ = *H*_c2_(||*ab*)/*H*_c2_(||*c*), which was determined from the in-field resistivity measurements. Compared with the LnFeAsO_1–*x*_F_*x*_ sample, the *γ*_*H*_ values of our SmFeAsO_1–*x*_H_*x*_ films are substantially smaller, a fact that originates from the increased dimensionality of the Fermi surface^36^. Specifically, F-doped *Ln*1111-type materials have two-dimensional Fermi surfaces containing hole and electron pockets, whereas H doping induces a three-dimensional hole pocket^36^. In fact, the resulting difference in anisotropy is rather remarkable at *T*/*T*_c_ ≈ 0.8, where F doping results in an anisotropy of ∼4–5 (refs. ^28,37^), which is halved in the highly doped regime by H doping. Such a reduction in anisotropy by doping has not been achieved in Y123, which has anisotropies of 5.5, 5.1 and 4.3 for underdoping^3^, optimal doping^38^ and overdoping^3^, respectively. Additionally, the *H*_c2_ anisotropy depends on temperature, which is not observed in Y123 data but has been seen in other iron-based superconductors^28,37,39–41^ (Fig. 5c).

We can also see the consequences of both reduced anisotropy and irradiation in our angle (*θ*)-dependent critical current density measurements, shown in Fig. 5d. Comparing the two pristine SmAsFeO_0.751_H_0.249_ and SmAsFeO_0.632_H_0.368_ films, the SmAsFeO_0.632_H_0.368_ film has a higher *J*_c_(*θ*) and a smaller anisotropy of *J*_c_ (${\gamma}_{J_{\mathrm{c}}} = J_{{\mathrm{c}}}^{\;ab}/J_{{\mathrm{c}}}^{\;c}$). This is due to the high *J*_c_^s.f.^ and small anisotropy of both *H*_c2_ (Supplementary Fig. 9a,b) and the irreversibility field (*H*_irr_; Supplementary Fig. 9c,d), a result of high hydrogen doping. Moreover, for the irradiated H-doped SmAsFeO_0.632_H_0.368_ film, an increase in *J*_c_ at all applied magnetic field orientations and an enhancement of *H*_irr_ are achieved (Supplementary Fig. 9c), which is a combined effect of high H doping, as well as defects aligned with the *c* axis and *ab* plane, introduced by proton irradiation. This is favourable over defects of a singular orientation, such as *c*-axis-aligned defects^1,42^, which produce a large peak at **H** ∥ *c*.

## Slowing vortex creep by reducing the Ginzburg parameter

While the maximum achievable *J*_c_ increases by increasing *J*_d_, our technique also reduces the vortex creep rates (*S*) in hole-doped Y123, 11-type, P-doped 122-type and electron-doped *Ln*1111-type films. For the *Ln*1111-type system, we specifically demonstrated this in Nd1111 samples of different doping levels as well as in a highly doped Sm1111 film. This is important because fast creep can decrease *J*_c_ and induced persistent currents. Our method not only increases *J*_c_, but also targets decreasing the Ginzburg number ${\mathrm{Gi}} \propto {T}_{{\rm{c}}\,}^{\,2}{\gamma }_{{{H}}\,}^{\,2}{\lambda }_{{{ab}}}^{\,4}/{\xi }_{{{ab}}}^{\,2}$, which parameterizes the impact of thermal fluctuations and positively correlates with creep rates^43^.

**Fig. 6:**
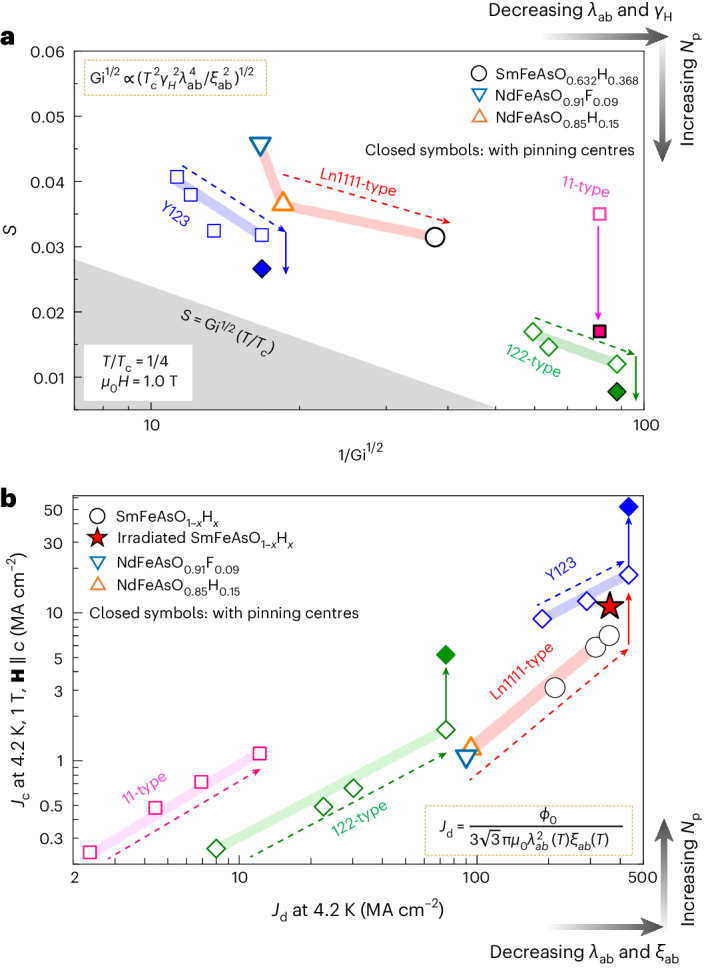
Dependence of *S* and *J*_c_ versus *J*_d_ after tuning various superconducting parameters. **a**, Flux creep rate *S* as a function of Gi^–1/2^ at *t* = *T*/*T*_c_ = 0.25 and 1 T for Y123 and three classes of iron-based superconductors. Data for the 11-type material (FeSe) is from the literature^50^. Increasing electron concentration decreases *S* for SmFeAsO_0.632_H_0.368_. This tendency was also observed for YBa_2_Cu_3_O_*y*_ with increased hole concentration^3^ and BaFe_2_(As_1–*x*_P_*x*_)_2_ with increased chemical pressure. The grey region indicates ranges of *S* below the predicted lower limit^43^, indicated by the equation *S*=Gi^1/2^(*T/T*_c_). **b**, In-field *J*_c_ as a function of the depairing current density *J*_d_ at 4.2 K and 1 T. Increasing carrier concentration by increasing electron and hole doping as well as chemical pressure enhances *J*_d_ in all displayed classes of superconductors. Additional enhancement can be achieved by the introduction of pinning centres. Closed symbols show data for samples containing vortex pinning centres (nanoparticle inclusions or irradiation-induced defects). Each coloured wide line connects the data within each class of material and the dashed arrows show how *S* (**a**) is decreased and *J*_c_ (**b**) is increased as our tuning parameter (doping or chemical pressure) increases. The coloured solid arrows show the changes in *S* (**a**) and *J*_c_ (**b**) upon adding vortex pinning centers (open symbols are data for pristine sample whereas solid symbols are for sample containing pinning centers). Source data

Figure 6a shows *S* versus 1/Gi^1/2^ at *T* = *T*_c_/4 and 1 T for various superconductors. First, reducing Gi in each material system reduces *S*. For example, the SmAsFeO_0.632_H_0.368_ film shows smaller *S* compared to that in the NdFeAsO_1–*x*_e_*x*_ films, which is due to the reduction of Gi, notably by reducing *λ*_*ab*_(0) and *γ*_*Η*_. Second, adding defects also slows creep. For technical reasons, we were unable to measure *S* for the irradiated SmAsFeO_0.632_H_0.368_ films, owing to a small sample volume; nevertheless, we expect reduced *S* as achieved in the other materials.

## Discussion

The standard method of increasing *J*_c_—focusing exclusively on introducing either weak collective pinning centres or strong pinning centres—typically has little effect on the penetration depth. A major advantage of our thermodynamic approach is that our ability to reduce the penetration depth *λ* has a dramatic effect on both Gi and *J*_d_ because Gi^1/2^ ≈ *λ*^2^ and *J*_d_ ≈ 1/*λ*^2^. Consequently, the advantages associated with reduced *λ* in SmFeAsO_1–*x*_H_*x*_ are further enhanced by the high calculated values for *J*_d_, achieved using this method, and we demonstrate substantial increases in *J*_d_ in many materials. Figure 6b therefore summarizes the main achievements of this work, displaying our measurements of the in-field *J*_c_ (*H* = 1 T, and **H** ∥ *c*) as a function of *J*_d_ at 4.2 K for Y123 and three classes of iron-based superconductors with varied pinning landscapes. Details of the calculation parameters for *J*_d_ and experimentally obtained *J*_c_ are shown in Supplementary Table 4. First, we see that *J*_c_ tends to positively correlate with *J*_d_. Note that SmFeAsO_1–*x*_H_*x*_ also reduced the creep rate (compared to the NdFeAsO_1–*x*_e_*x*_ films), which may have contributed to the increased *J*_c_ in this material. Second, we see that for the pristine SmFeAsO_1–*x*_H_*x*_ films, *J*_c_(4.2 K) at 1 T and **H** ∥ *c* increases from 3.1 to 7.0 MA cm^–2^, correlated with an increase in *J*_d_ tuned by the electron concentration. Third, at 4.2 K and 1 T for **H** ∥ *c*, the combination of tuning *J*_d_ and adding a high density of nano-defects produces an in-field *J*_c_ of 11 MA cm^–2^ and a corresponding *J*_c_/*J*_d_ of 3.1% (where *J*_d_(4.2 K) = 360 MA cm^–2^) that is comparable to the pristine Y123 conductor^44^.

In the introduction, we discussed the coincidence between the peak in *J*_d_ and a QCP in Y123. Such correspondence is unsurprising. Because *J*_d_(*T*) ≈ 1/*λ*^2^ ∝ *n*_s_/m* and if the *n*_s_ diverges around the QCP, there may be a precipitous dip in the doping-dependent *λ*, engendering an apex in *J*_d_, therefore enabling an unprecedentedly high *J*_c_. However, the behaviour of *λ* at the QCP, or more generally with doping, depends on the relative rate of change of the effective mass versus *n*_s_, and this behaviour does not appear to be universal. Consistent with our study, a dip in *λ* was captured in a hole-doped cuprate (related to pseudo-gap formation)^45^. On the other hand, in BaFe_2_(As_1–*x*_P_*x*_)_2_ (ref. ^46^) and Ba(Fe_1–*x*_Co_*x*_)_2_As_2_ (ref. ^47^), a sharp peak in *λ* appears at the QCP. Regarding *Ln*1111-type superconductors, although a QCP has been found in LaFeAsO_1–*x*_H_*x*_ (ref. ^48^) and evidence of a possible QCP in NdFeAsO_1–*x*_H_*x*_ has been discussed^30^, no QCP has yet been found in other *Ln*1111-type materials. Given the potential for extremely high *J*_c_ values at QCPs, further studies of the relationship between QCPs and *J*_d_ are warranted.

In summary, using a combined approach of tuning *J*_d_ by controlling *λ* and *ξ* through the tuning of the carrier density and enhancing flux pinning by irradiation, we substantially boost *J*_c_ in SmFeAsO_1–*x*_H_*x*_ films. Our films showed very high in-field *J*_c_ values in iron-based superconductors. This increase should by no means be the maximum achievable *J*_c_ in SmFeAsO_1–*x*_H_*x*_; for Y123, ∼15% of *J*_d_ has been reported in a field of 3 T, and 32.4% of *J*_d_ has been reported at self-field^3^, near the predicted maximum that is achievable with core pinning^4–6,49^. Consequently, the remarkably high *J*_d_ of 415 MA cm^–2^ that we achieved sets a new ceiling for *J*_c_ in SmFeAsO_1–*x*_H_*x*_ films. Further studies are warranted to fully optimize pinning in this material. Our methods also reduce the effective mass anisotropy *γ* and Gi, resulting in slower vortex creep. We have therefore demonstrated that this combined approach is effective in improving the performance of superconductors of different families: hole-doped cuprates (Y123)^3^ and iron-based superconductors (electron-doped *Ln*1111-type, Te-doped 11-type and chemically pressured P-doped 122-type^3^).

## Methods

### Sample preparation

The undoped SmFeAsO epitaxial films on MgO(001) single crystals were grown by pulsed laser deposition to a thickness of 50 nm. Film thickness was measured using a step profiler and confirmed using cross-sectional transmission electron microscopy. Doped SmFeAsO_1–*x*_H_*x*_ epitaxial thin films were prepared by topotactic chemical reaction between the undoped SmFeAsO epitaxial film and CaH_2_ powder. More details regarding the growth method, describing the pulsed laser deposition process and thermal annealing, can be found in ref. ^20^. The NdFeAsO_1–*x*_H_*x*_ and NdFeAsO_1–*x*_F_*x*_ epitaxial films were grown on MgO(001) single crystals using molecular beam epitaxy^24^. The Y123 nanocomposite films were grown epitaxially from metal organic solutions^3^. Lastly, both the BaFe_2_(As_1−*x*_P_*x*_)_2_ and the FeSe_1–*x*_Te_*x*_ (ref. ^15^) epitaxial films were deposited using pulsed laser deposition.

Ion irradiation of the SmFeAsO_1–*x*_H_*x*_ thin films was performed using the 400 kV ion implanter at the Takasaki Ion Accelerators for Advanced Radiation Application facility of the Takasaki Institute for Advanced Quantum Science (QST). Proton beams of energy 150 keV were directed at the film surfaces at normal incidence and a fluence of 5.3 × 10^14^ ions cm^–2^.

### Transport properties in magnetic fields

The films were patterned using a pulsed fibre laser into bridges of ∼30 μm width. The temperature dependence of the resistivity (*ρ*) was measured by a four-probe method in the temperature range of 4–300 K using a Physical Property Measurement System with a superconducting magnet generating a field **H** up to 9 T and in a 25 T superconducting magnet at Tohoku University. The critical current density was determined using a 1 μV cm^−1^ criterion. The resistivity criterion for determining *H*_c2_ is 80% of the temperature dependence of the normal state resistivity (*ρ*_N_(*T*)) which is extrapolated from the available *ρ*_N_(*T*) up to just above *T*_c_^onset^. We compared our data in Fig. 5c to data reported by Hanzawa et al.^22^ and thus chose the same metric for extracting *H*_c2_. Similarly, *H*_irr_ was determined using 1% of *ρ*_N_(*T*). Hall measurements were conducted in a magnetic field of 9 T, and magnetization studies were performed using a superconducting quantum interference device (SQUID) magnetometer to characterize the temperature and field dependence of *S*.

## Online content

Any methods, additional references, Nature Portfolio reporting summaries, source data, extended data, supplementary information, acknowledgements, peer review information; details of author contributions and competing interests; and statements of data and code availability are available at 10.1038/s41563-024-01952-7.

## Supplementary information

Supplementary Information Supplementary Figs. 1–9 and Tables 1–3.

## Source data

Source Data Fig. 4 Analysed data used to plot Fig. 4a–f.

Source Data Fig. 5 Analysed data used to plot Fig. 5a–d.

Source Data Fig. 6 Analysed data used to plot Fig. 6a,b.

## Data Availability

The data that support the findings of this study are available from the corresponding author on reasonable request. Source data are provided with this paper.
