# Supplementary material for: Quadrupling the depairing current density in the iron-based superconductor SmFeAsO1–xHx
Source: Nat Mater. 2024 Jul 18;23(10):1370–8. doi: 10.1038/s41563-024-01952-7 (PMC11442304; doi:10.1038/s41563-024-01952-7)
Supplement: Supplementary file 1 — Supplementary Figs. 1–9 and Tables 1–3. [file 41563_2024_1952_MOESM1_ESM.pdf]

nature materials

Supplementary information

https://doi.org/10.1038/s41563-024-01952-7

Quadrupling the depairing current density in the iron-based superconductor $SmFeAsO_{1-x}H_x$

In the format provided by the authors and unedited

Contents

(1) Calculation of zero-temperature depairing current density for various superconducting materials---------------------2

(2) H concentration for $SmFeAsO_{1-x}H_x$ films--3

(3) Temperature dependence of resistivity and self-field critical current density for $SmFeAsO_{1-x}H_x$ films-----------------4

(4) The Hall coefficient for $SmFeAsO_{1-x}H_x$ films--5

(5) Estimation of $H_{c2}(0)$ in $SmFeAsO_{1-x}H_x$ films---7

(6) Calculation of the penetration depth using the measured resonant frequency--11

(7) Field dependence of J_c for pristine and irradiated $SmFeAsO_{0.632}H_{0.368}$ films--12

(8) Temperature dependence of the upper critical field and irreversibility field for $SmFeAsO_{1-x}H_x$ films----------------13

(9) The parameters for the calculation of J_d at 4.2 K for different superconductors--14

References--15

(1) Calculation of zero-temperature (T = 0 K) depairing current density for various superconducting materials

In order to calculate the depairing current density, J_d at T= 0 K, for different superconducting materials, we applied the Tinkham formula within Ginzburg-Landau theory $J_d(T=0)=\frac{\phi_0}{3\sqrt{3}\pi\mu_0\lambda_{ab}^2(0)\xi_{ab}(0)}$ [10], where $\lambda_{ab}(0)$ and $\xi_{ab}(0)$ are the penetration depth and coherence length, respectively. The values for $\lambda_{ab}(0)$ and $\xi_{ab}(0)$ used in the calculation of $J_d(0)$ are listed in **Table S1**.

Table S1: Critical temperature T_c, coherence length $\lambda_{ab}(0)$, and penetration depth $\lambda_{ab}(0)$ used to calculate $J_d(0)$ for $HgBa_2CuO_{4+\delta}$ [12, 13], $YBa_2Cu_3O_y$ [3, 17] ,$FeSe_{1-x}Te_x$ [15] and $SmFeAsO_{1-x}H_x$. Zero-temperature values for the latter two parameters are noted. In the Table S1, p is the carrier concentration in the Cu-based superconductors.

Materials	T_c (K)	$\xi_{ab}(0)$ (nm)	$\lambda_{ab}(0)$ (nm)	$J_d(0)$ (MA /cm²)
$HgBa_2CuO_{4+\delta}$				
p = 0.061	48.90 [13]	2.09 [13]	262 [12]	70.3
p = 0.078	65.48 [13]	2.45 [13]	223 [12]	82.7
p = 0.093	71.55 [13]	2.66 [13]	190 [12]	105.0
p = 0.100	75.59 [13]	2.72 [13]	178 [12]	117.0
p = 0.117	85.70 [13]	2.59 [13]	155 [12]	162.0
p = 0.133	92.58 [13]	2.41 [13]	141 [12]	210.4
p = 0.154	95.41 [13]	2.17 [13]	138 [12]	243.9
p = 0.174	92.21 [13]	1.86 [13]	150 [12]	240.9
p = 0.183	88.13 [13]	1.79 [13]	158 [12]	225.6
p = 0.188	86.11 [13]	1.80 [13]	166 [12]	203.2
p = 0.211	74.38 [13]	2.14 [13]	197 [12]	121.4
$YBa_2Cu_3O_y$				
p = 0.064	23.64 [17]	3.36 [17]	194 [3]	79.7
p = 0.078	44.27 [17]	2.57 [17]	181 [3]	119.7
p = 0.102	58.83 [17]	3.30 [17]	163 [3]	115.0
p =0 .109	61.25 [17]	4.19 [17]	158 [3]	96.4
p = 0.120	67.32 [17]	4.19 [17]	149 [3]	108.4
p = 0.136	78.64 [17]	3.06 [17]	139 [3]	170.5
p = 0.141	82.69 [17]	2.71 [17]	136 [3]	201.1
p = 0.151	90.77 [17]	2.17 [17]	129 [3]	279.1
p = 0.162	93.62 [17]	1.71 [17]	125 [3]	377.3
p = 0.174	92.80 [17]	1.52 [17]	120 [3]	460.5
p = 0.181	90.37 [17]	1.50 [17]	116 [3]	499.4
p = 0.191	85.52 [17]	1.56 [17]	114 [3]	497.2
p = 0.206	74.60 [17]	1.85 [17]	119 [3]	384.8
$FeSe_{1-x}Te_x$ film				
x = 0.00	9.53 [15]	3.85 [15]	685 [15]	5.6
x = 0.20	21.58 [15]	2.40 [15]	499 [15]	16.9
x = 0.40	16.07 [15]	2.05 [15]	673 [15]	10.9
x = 0.50	13.02 [15]	2.12 [15]	774 [15]	7.9
$SmFeAsO_{1-x}H_x$ film				
x = 0.249	43.93	1.81	150	247.5
x = 0.339	46.51	1.52	135	363.9
x = 0.368	46.55	1.46	129	414.9

(2) H concentration for $SmFeAsO_{1-x}H_x$ films

We quantitatively analyzed H concentrations (x in $SmFeAsO_{1-x}H_x$) in our samples by Secondary Ion Mass Spectroscopy (SIMS) using a $SmFeAsO_{0.4}H_{0.6}$ polycrystalline bulk sample as a reference. **Figure S1(a)** shows SIMS depth profiles for samples No. 1 ($J_c^{s.f.}$(4.2K)=4.11 MA/cm^2), No. 2 ($J_c^{s.f.}$(4.2K)=7.12 MA/cm^2), No. 3 ($J_c^{s.f.}$(4.2K)=9.22 MA/cm^2) and No. 4 ($J_c^{s.f.}$(4.2K)=7.03 MA/cm^2), respectively. **Figure S1(b)** is a summary of x values; given that x varies with thickness, the compositions allocated to each sample are an average value from thicknesses ranging from 10 to 42 nm. The x values were 0.25 for No. 1, 0.339 for No. 2, 0.368 for No. 3 and 0.397 for No. 4.

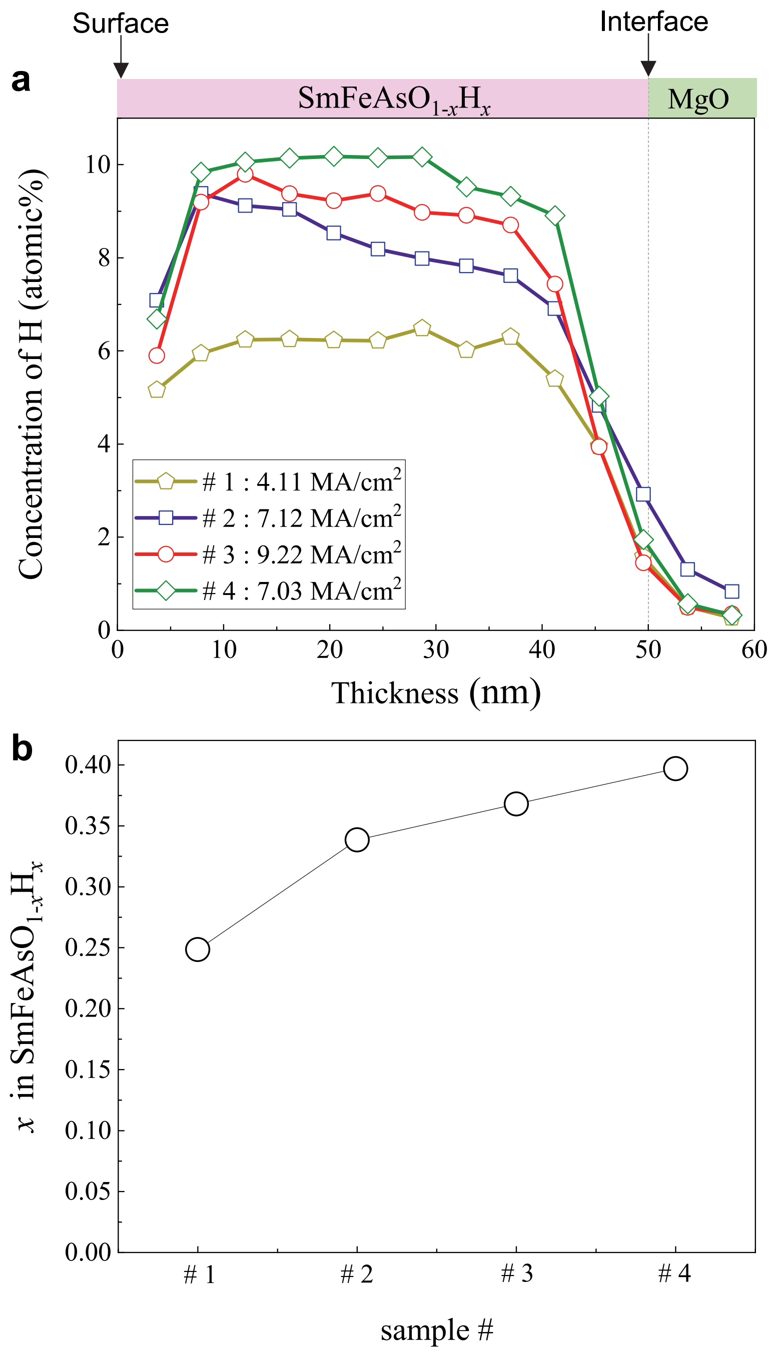

Figure S1: (a) SIMS profiles of #1, #2, #3 and #4 as a function of depth from the surface, i.e., a depth of 0 nm denotes the top of the film surface. **(b)** x for all four films (Nos. 1–4).

(3) Temperature dependence of resistivity and self-field critical current density for $SmFeAsO_{1-x}H_x$ films

To study the influence of irradiation on T_c in the pristine and irradiated $SmFeAsO_{0.632}H_{0.368}$ films, we measured the temperature dependence of the resistivity, shown in **Fig. S2 (a)**. The pristine and irradiated films exhibit almost the same critical temperatures T_c (only a 0.4 K difference). **Fig. S2(b)** shows the *E*-*J* curve for various H-doped Sm1111 films. Although the pristine and irradiated films have almost the same T_c, the $J_c^{s.f.}$ of the irradiated $SmFeAsO_{0.632}H_{0.368}$ film is over 1.75 times higher than that of the pristine film.

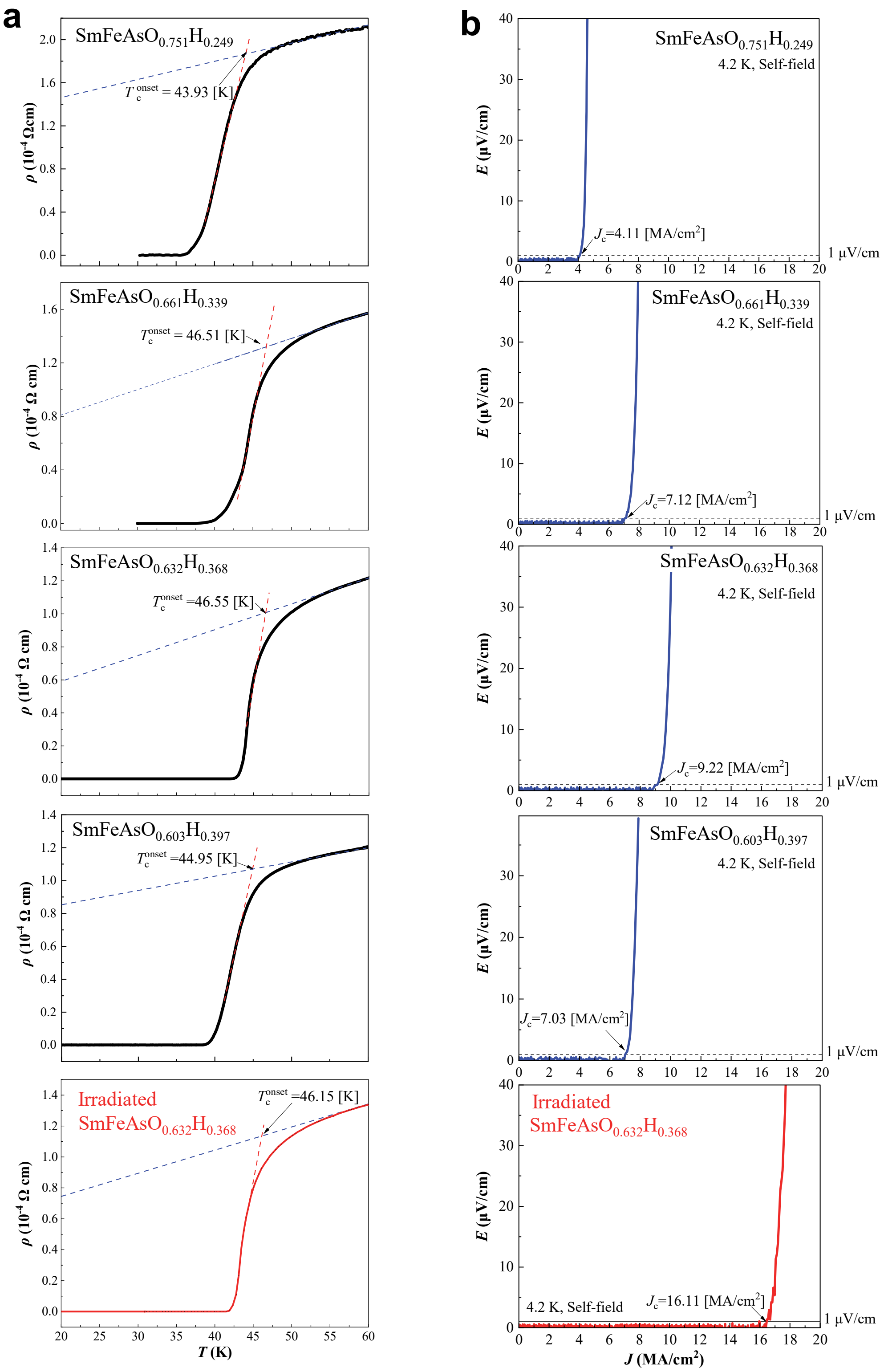

Figure S2: **(a)** Temperature T dependent resistivity ρ for pristine and irradiated films. **(b)** E as a function of current density at 4.2 K and self-field for pristine and irradiated films.

(4) The Hall coefficient for $SmFeAsO_{1-x}H_x$ films

Fig. S3(a) shows the Hall coefficient R_H as a function of T measured in a superconducting magnet at ±9 T for various hydrogen-doped pristine and irradiated films. R_H for the polycrystalline $NdFeAO_{0.94}F_{0.06}$ sample, from Ref. [23], is also shown. The R_H for all samples were negative, indicating electrons as that the dominant carriers. If only one band contributes to charge transport, then $R_H = -1/ne = -1/(n_e - n_h)e$ relates the carrier density n to the Hall coefficient from Hall effect measurements, where n, n_e, and n_h are total carrier, electron, and hole densities, respectively. **Fig. S3(b)** shows the temperature dependence of n determined from the R_H versus T data. Compared with NdFeAs(O,F) [23] having a higher T_c of 50.5 K, the carrier densities of the $SmFeAsO_{1-x}H_x$ films (T_c~46.5 K) were larger and we see that doping increases the carrier density. It is worth noting that the pristine and irradiated samples show similar n, as shown in **Fig. S3(b)**.

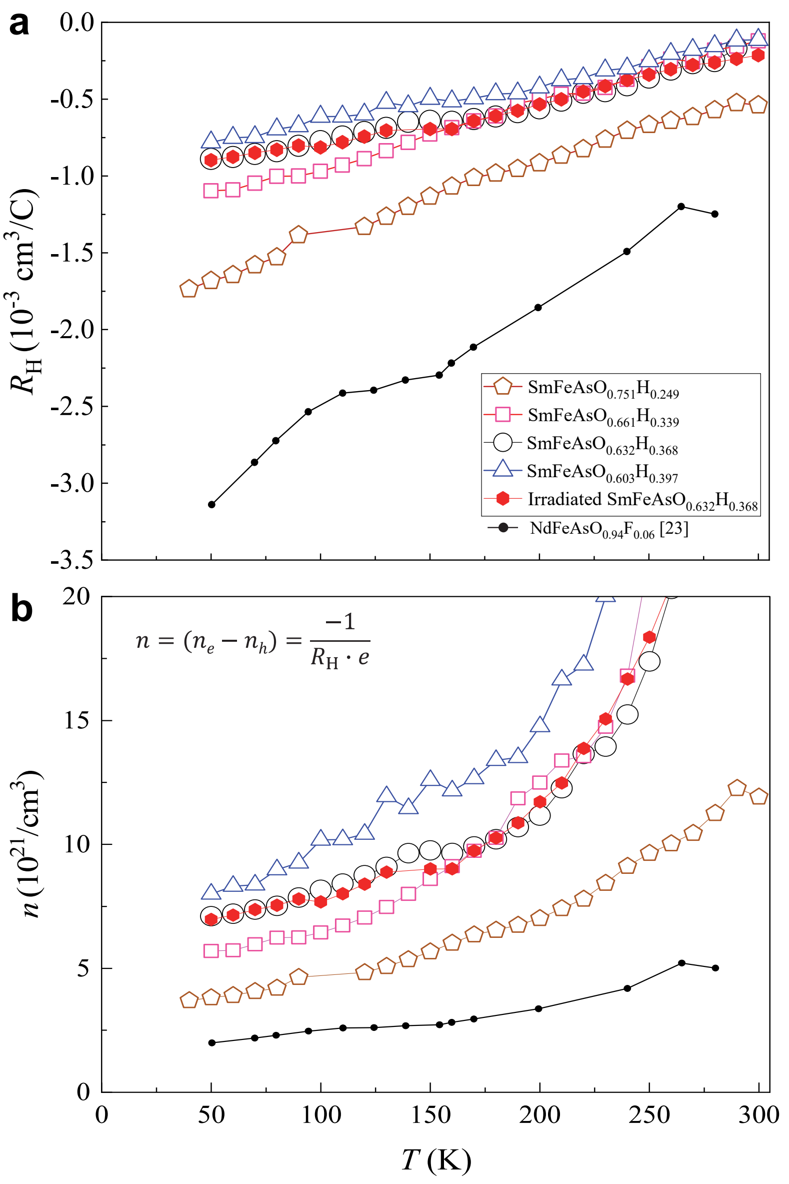

Figure S3: (a) Hall coefficient R_H as a function of T measured in a magnetic field of ±9 T for various films. **(b)** the temperature dependence of n determined from the R_H versus T data.

Table S2: Important sample parameters (irradiation condition, in-plane crystallinity, T_c^{onset}, n^{50K} and $J_c^{s.f.}$) for pristine and irradiated $SmFeAsO_{1-x}H_x$ films. In-plane crystallinity was evaluated using the full width at half maximum values of the 200 reflection of $SmFeAsO_{1-x}H_x$ films.

Sample #	Film material	substrate	Irradiation condition	Thickness (nm)	In-plane crystallinity (degree)	T_c^{onset} (K)	n^{50K} (10^{21} /cm^3)	$J_c^{s.f.}$@4.2K (MA /cm^2)
	**Pristine film**							
# 1	$SmFeAsO_{0.751}H_{0.249}$	MgO	N/A	90	1.40	43.93	3.70	4.11
# 2	$SmFeAsO_{0.661}H_{0.339}$	MgO	N/A	50	1.35	46.51	5.69	7.12
# 3	$SmFeAsO_{0.632}H_{0.368}$	MgO	N/A	50	1.35	46.55	7.10	9.22
# 4	$SmFeAsO_{0.603}H_{0.397}$	MgO	N/A	50	1.35	44.95	7.99	7.03
	**Irradiated film**							
# 5	Irradiated $SmFeAsO_{0.661}H_{0.339}$	MgO	150 keV H+, 1.16x10^{15} /cm^2	50	1.38	46.29	-	12.02
# 6	Irradiated $SmFeAsO_{0.632}H_{0.368}$	MgO	150 keV H+, 1.16x10^{15} /cm^2	50	1.39	46.15	6.97	16.11

(5) Estimation of $H_{c2}(0)$ in $SmFeAsO_{1-x}H_x$ films

Figs. S4(a)-(e) show the temperature and applied magnetic field dependent resistivity ρ in the various H-doped pristine Sm1111 films and an irradiated $SmFeAsO_{0.632}H_{0.368}$ film. Here, the magnetic fields $\mu_0 H$ is applied parallel to the c axis.

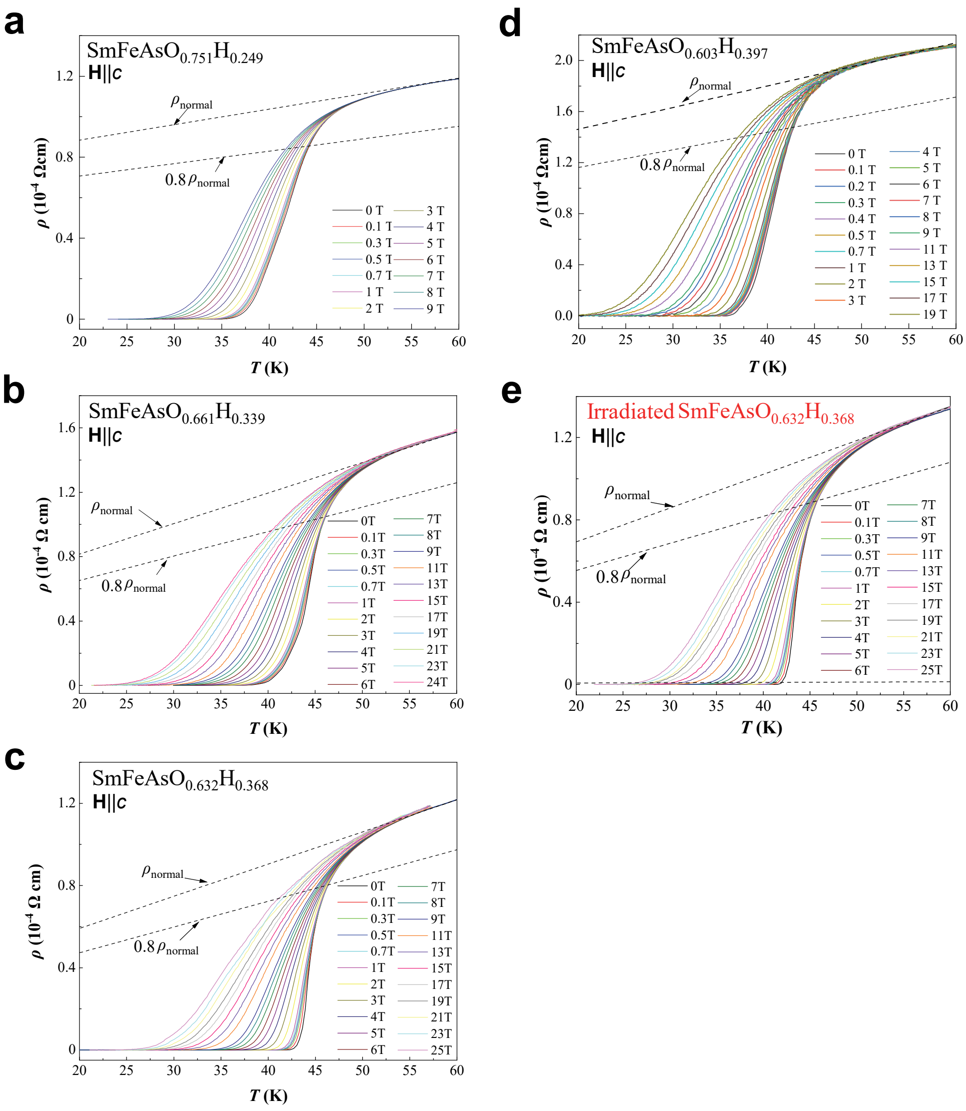

Figure S4: Temperature dependence of resistivity ρ in various magnetic fields μ_0H applied parallel to the c axis for pristine $SmFeAsO_{1-x}H_x$ films with **(a)** x=0.25, **(b)** 0.339, **(c)** 0.368, **(d)** 0.397 and **(e)** irradiated $SmFeAsO_{0.632}H_{0.368}$ film.

The temperature-dependence of H_{c2} depends on the band structure (single or multiband) and scattering (e.g. dirty or clean limit, inter- and intra-band scattering). For example, WHH formula [26] is most commonly applied for calculating $H_{c2}(0)$, though in its original form strictly models the case of single-band superconductors. Iron-based superconductors, however, are multiband with disconnected Fermi surfaces and are typically in the clean limit [27]. Models of $H_{c2}(T)$ derived for iron-based superconductors extend from generalizations of the WHH model, considering unconventional symmetry of the order parameter, inter- and intra-band Cooper pairing, as well as inter- and intra-band impurity scattering [27]. As noted in Refs. [27, 51], most iron-based superconductors, including *Ln*1111 type, should be in the clean limit (electron mean free path $l_{ab} >> \xi_{ab}$). To determine whether our Sm1111 films are in the dirty- or clean-limit, we calculated the electron mean free path (l_{ab}) in the normal state. As an example, for the $SmFeAsO_{0.632}H_{0.368}$ film at 50 K, using a zero-temperature resistivity of $\rho(0)$=45.0 mΩcm and a carrier density of n= 7.01×10^{27} m^{-3}, the *ab*-plane mean free path is estimated as $l_{ab} = \hbar(3\pi^2)^{1/3}/\rho(0)e^2n^{2/3}$=7.71 nm. Since for all of our films, the mean free paths l_{ab} are larger than the coherence lengths $\xi_{ab}(0)$ (=2.25-2.60 nm) reported in the Ln1111 system [24, 28 and 29], the properties of $SmFeAsO_{1-x}H_x$ films are indeed within the clean limit. To estimate the zero-temperature critical field $\mu_0H_{c2}(0)$ for **H**||*c*, we also fitted $\mu_0H_{c2}(T)$ with **H**||*c* using the clean limit two-band Gurevich model [27, 51]. To simplify the fitting process, we fixed the intra-band coupling constants to $\lambda_{11} = \lambda_{22} = 0$ and the inter-band coupling constants to $\lambda_{12} = \lambda_{21} = 0.5$ [52] where λ_{mn} is the superconducting coupling constant between the *m*th and *n*th bands. Further details regarding the fitting parameters can be found in previous reports [27, 51]. In **Fig. S5**, we show the clean limit two-band model with $\eta=(v_2/v_1)^2$ where v_m is the Fermi velocity in the *m*th band and the Pauli paramagnetic effect $\alpha_1 = \frac{4\mu\phi_0T_c}{\hbar v_1^2} = \frac{\pi k_B T_c m}{E_F m_0}$ where E_F is the Fermi energy, k_B is the Boltzmann constant and ϕ_0 is the magnetic flux quantum. For all compositions, we found that the results can be reproduced with η=0.29-0.37 and α_1=0.16-0.27 for **H**||*c*. As shown in **Fig. S5**, the clean limit two-band Gurevich model agrees well with most of our experimental data. The obtained $H_{c2}^{\text{two-band}}(0)$, calculated values of $\xi_{ab}(0)$, and resulting $J_d(0)$ are summarized in **Table S3**. For comparison, the estimated $H_{c2}(0)$ using the Ginzburg-Landau formula [10] $H_{c2}^{GL}(0) = T_c|dH_{c2}/dT|_{T_c}$ and clean limit Werthamer–Helfand–Hohenberg (WHH) model $H_{c2}^{orb}(0) = 0.73T_c|dH_{c2}/dT|_{T_c}$ [26] are also listed in **Table S3**. In this work, using the obtained $H_{c2}^{\text{two-band}}(0)$ from **Fig. S5**, the $\xi_{ab}(0)$ is calculated from the Ginzburg-Landau formula: $\mu_0H_{c2}(0)=\phi_0/2\pi\xi_{ab}(0)^2$. As a result, we see that $J_d(0)$ for the $SmFeAsO_{0.632}H_{0.368}$ film is nearly quadruple that of the $SmFeAsO_{0.8}H_{0.2}$ [29, 33] and $NdFeAsO_{0.88}F_{0.12}$ [28, 32].

As is evident in the insets in **Figs. S6(c)** and **S6(e)**, $H_{c2}(T)$ for the pristine and irradiated $SmFeAsO_{0.632}H_{0.368}$ films show an interesting downward curvature near T_c, affecting a tight range of temperatures only within 2% of T_c. Although most of our experimental $H_{c2}(T)$ data fit well to the clean limit two-band curve, the downward curvature near T_c in the films with x=0.368 notably deviates. According to Refs. [27, 51] by Gurevich et al., the slope of H_{c2} near T_c depends on the enhanced effective mass m/m_0 and T_c/E_F ratios as $\left|\frac{dH_{c2}}{dT}\right|_{T_c} = \frac{1.8T_c m}{(1+\eta)E_F m_0}$.

From $\alpha = \frac{\pi k_B T_c m}{E_F m_0}$ and $\left|\frac{dH_{c2}}{dT}\right|_{T_c} = \frac{1.8T_c m}{(1+\eta)E_F m_0}$, we see that $\alpha \propto \frac{T_c m}{E_F m_0} \propto \left|\frac{dH_{c2}}{dT}\right|_{T_c}$ which indicates that the $SmFeAsO_{0.632}H_{0.368}$ film is in the very strong Pauli limit near T_c compared to the other films. Moreover, this interesting phenomenon is suggestive of the influence of multiband behavior rather than simply two-band behavior. The former is likely related to the variation of the m/m_0 and T_c/E_F ratios by changing the H-doping level in $SmFeAsO_{1-x}H_x$ films. In term of the latter, in general, there are five bands across the Fermi surface in the *Ln*1111 system. Further theoretical and experimental investigation is warranted.

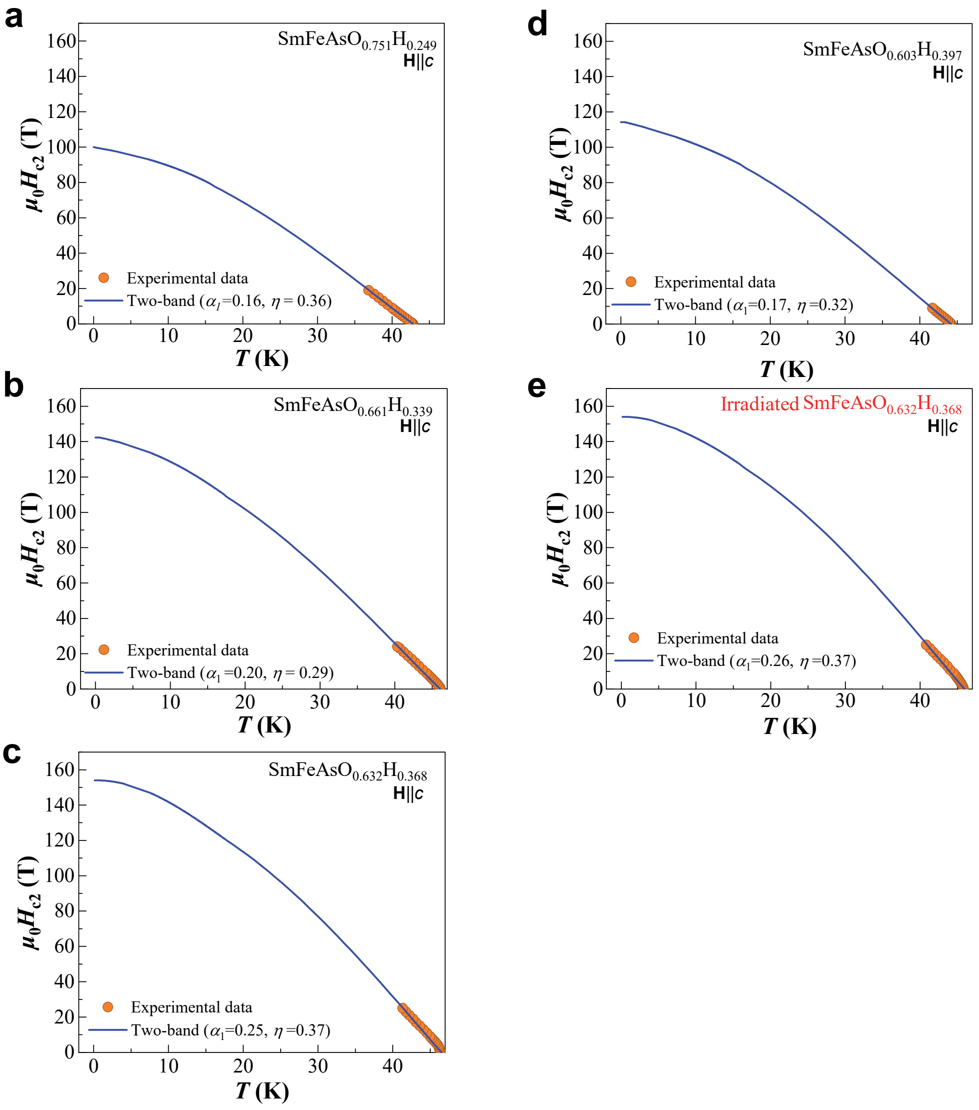

Figure S5: Temperature dependence of the upper critical field $\mu_0 H_{c2}(T)$ with **H**||*c* for $SmFeAsO_{1-x}H_x$ films with various *x*. The solid lines are the clean limit two-band Gurevich model [27, 51] predictions with best fitting parameters.

Table S3 Parameters used in the calculation of J_d at 0 K for pristine and irradiated films.

Film material	$\mu_0 H_{c2}^{\text{two-band}}(0)$ (T)	$\mu_0 H_{c2}^{\text{orb}}(0)$ (T)	$\mu_0 H_{c2}^{\text{GL}}(0)$ (T)	$\xi_{ab}(0)$ ** (nm)	$\lambda_{ab}(0)$ (nm)	$J_d(0)$ (MA /cm^2)
$NdFeAsO_{0.88}F_{0.12}$ [28]	67.6	67.5	92.4	2.21	200 [34]	114.0
$SmFeAsO_{0.8}F_{0.20}$ [29]	52.0*	52.1	71.4	2.52	190 [35]	111.0
Pristine film						
$SmFeAsO_{0.75}H_{0.25}$	100.0	99.4	136.2	1.81	150	247.5
$SmFeAsO_{0.661}H_{0.339}$	142.1	142.3	195.0	1.52	135	363.9
$SmFeAsO_{0.632}H_{0.368}$	154.0	165.6	226.8	1.46	129	414.9
$SmFeAsO_{0.603}H_{0.397}$	114.2	114.6	157.1	1.70	-	-
Irradiated film						
Irradiated $SmFeAsO_{0.632}H_{0.368}$	156.5	166.4	228.0	1.45	129	417.8

*$\mu_0 H_{c2}^{\text{two-band}}(0)$ is estimated with dirty limit two-band model [29].

**$\xi_{ab}(0)$ is estimated with $\mu_0 H_{c2}(0)=\phi_0/2\pi\xi_{ab}(0)^2$ for $\mu_0 H_{c2}^{\text{two-band}}(0)$.

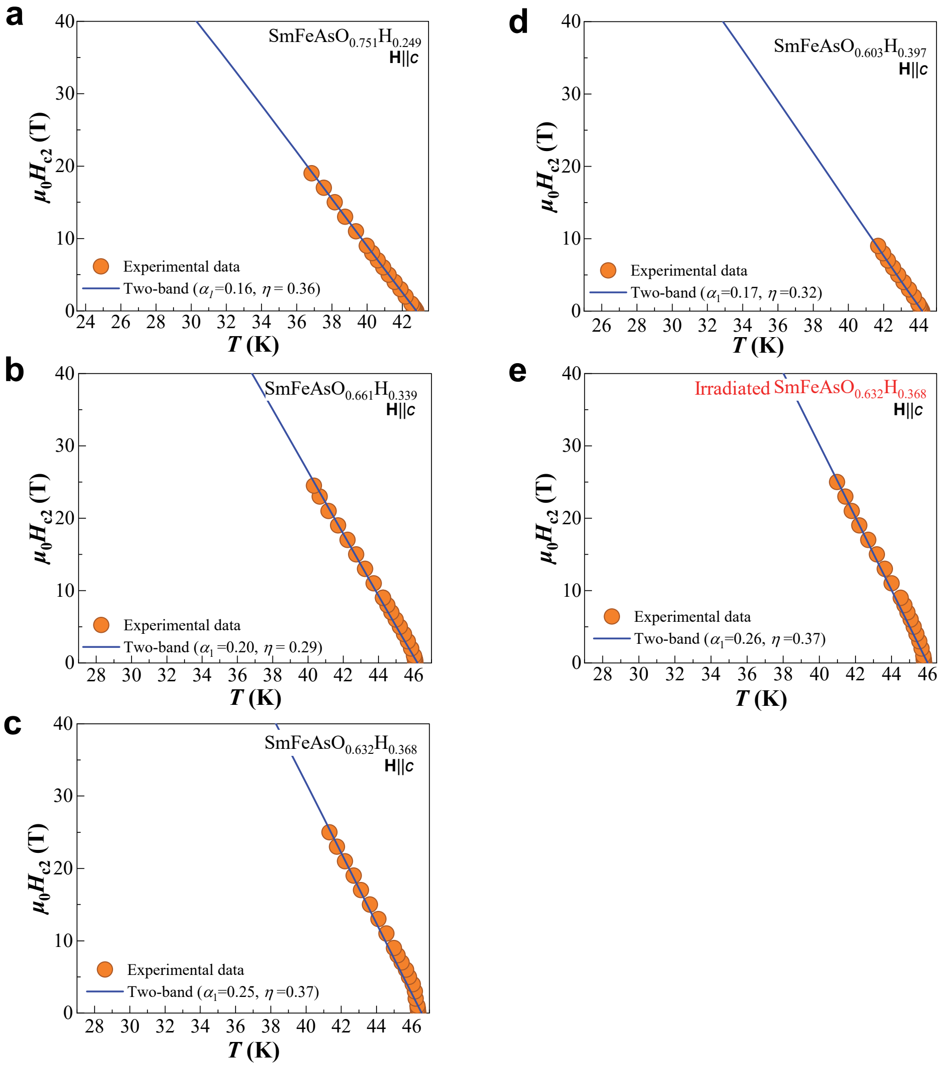

Figure S6: An enlargement of $\mu_0 H_{c2}(T)$ at **H**||c with clean-limit two band model fitting [27] for $SmFeAsO_{1-x}H_x$ films with various x.

(6) Calculation of the penetration depth using the measured resonant frequency

We designed and fabricated coplanar waveguide (CPW) resonators of $SmFeAsO_{1-x}H_x$ films on MgO substrates, and measured the temperature dependence of the resonant frequency f_0. The substrate size was 7.5 ×5.0 × 0.5 mm. An electromagnetic simulator (Sonnet EM software) was used to design the resonator. **Fig. S7 (a)** shows a picture of the fabricated CPW resonator. We designed a half-wavelength straight line resonator using photolithography and Ar^+ ion milling, as shown in the lower panel of **Fig. S7(a)**. The top panels of **Fig. S7(b)** and **(c)** show the temperature dependence of the resonance frequency for resonators with x=0.249 and 0.368, respectively. The resonant frequency f_0 can be expressed as

$$f_0 = \frac{1}{2l\sqrt{LC}} \quad (1)$$

where l is the length of the coplanar resonator, L is the total inductance per unit length, and C is the capacitance per unit length. For a superconducting coplanar waveguide, L consists of two terms:

$$L = L_\mathrm{m} + L_\mathrm{k} \quad (2)$$

$$L_\mathrm{m} = \frac{\mu_0 K(k\prime)}{4K(k)} \quad (3)$$

$$L_\mathrm{k} = \frac{\mu_0 g(s,w,d)}{dw}\lambda^2 \quad (4)$$

where L_m is the magnetic inductance which is determined by its geometry, L_k is the kinetic inductance which comes from the response of the superfluid, $K(k)$ is the complete elliptic integral of the first kind with a modulus $k = w/(w+2s)$, μ_0 is the vacuum permeability, d is the thickness of the resonator, w is the width of the center conductor, s is the gap between the center conductor and the ground, and $g(s, w, d)$ is a geometric factor. For our CPW resonators, d = 30 nm, w =100 μm, and s =100 μm.

From Eqs. (1) to (4), λ can then be expressed as follows: $$\lambda(T) = \sqrt{\frac{dw}{\mu_0 g}\left(\frac{1}{4l^2 C f_0^2} - L_\mathrm{m}\right)}. \quad (5)$$

Further details regarding the calculation of λ from f_0 have been published elsewhere [15, 53]. **Fig. S7(b)** shows the calculated temperature dependence of λ using the experimentally obtained f_0 for $SmFeAsO_{1-x}H_x$ films with different H concentration.

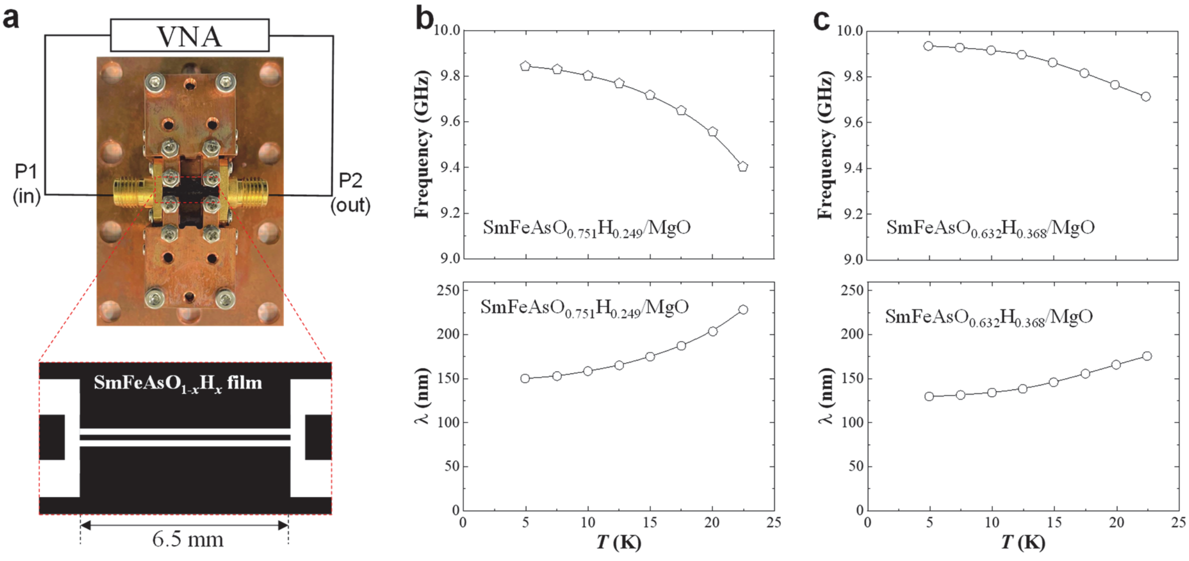

Figure S7: (a) Optical image and schematic of the fabricated CPW resonator. **(b)** and **(c)** Top panels: Temperature dependence of the resonance frequency (f_0) of the coplanar resonators, Bottom panels: Temperature dependence of the calculated penetration depth (λ) of the $SmFeAsO_{1-x}H_x$ films with different x.

(7) Field dependence of J_c for pristine and irradiated $SmFeAsO_{0.632}H_{0.368}$ films

Fig. S8 displays the raw J_c-$\mu_0 H$ data at both **H**||*c* and **H**||*ab* used to calculate the ratio of $J_c^{irraditaed}$ to $J_c^{pristine}$ shown in the inset of **Fig. 5b**.

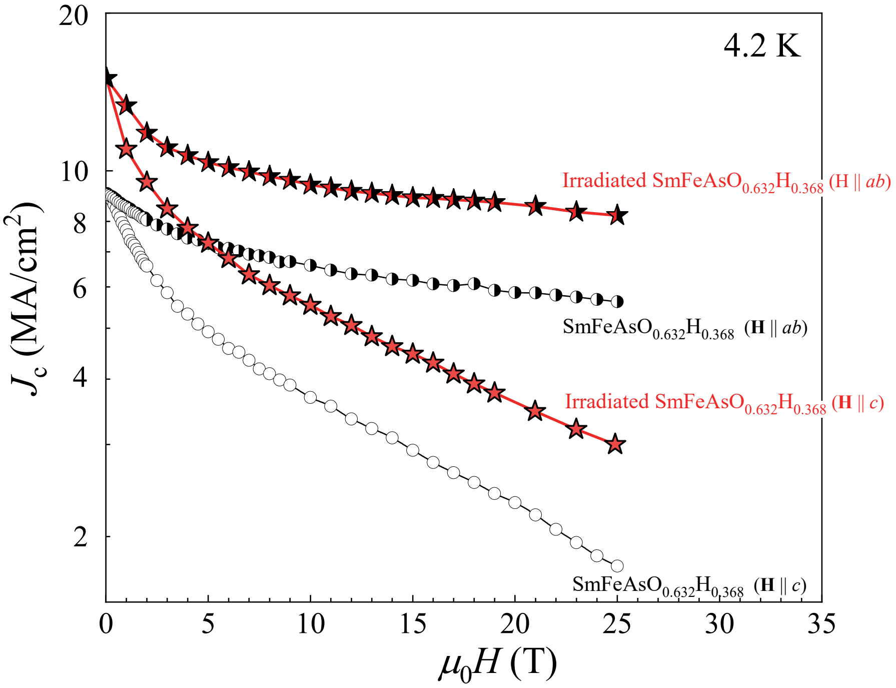

Figure S8: Field dependence of J_c at 4.2 K for the pristine and irradiated $SmFeAsO_{0.632}H_{0.368}$ films.

(8) Temperature dependence of the upper critical field and irreversibility field for $SmFeAsO_{1-x}H_x$ films

Fig. S9(a) shows is the temperature dependence of the upper critical field (H_{c2}) for Sm1111 thin films with x=0.249 and x=0.368. For x=0.368, H_{c2} is also plotted after irradiation. The corresponding H_{c2} anisotropy γ_H as a function of reduced temperature $t = T/T_c$ is shown in **Fig. S9(b)**. As can be seen, γ_H of Sm1111 (x=0.368) is lower than that of Sm1111 (x=0.249). **Fig. S9(c)** shows the temperature dependence of the irreversibility field (H_{irr}) for the H-doped Sm1111 films shown in **Fig. S9(a)**. After irradiation, H_{irr} of Sm1111 (x=0.368) was enhanced for both crystallographic directions. The H_{irr} anisotropy γ_{Hirr} of Sm1111 (x=0.368) is lower than that of Sm1111 (x=0.249). γ_{Hirr} was almost unchanged by irradiation.

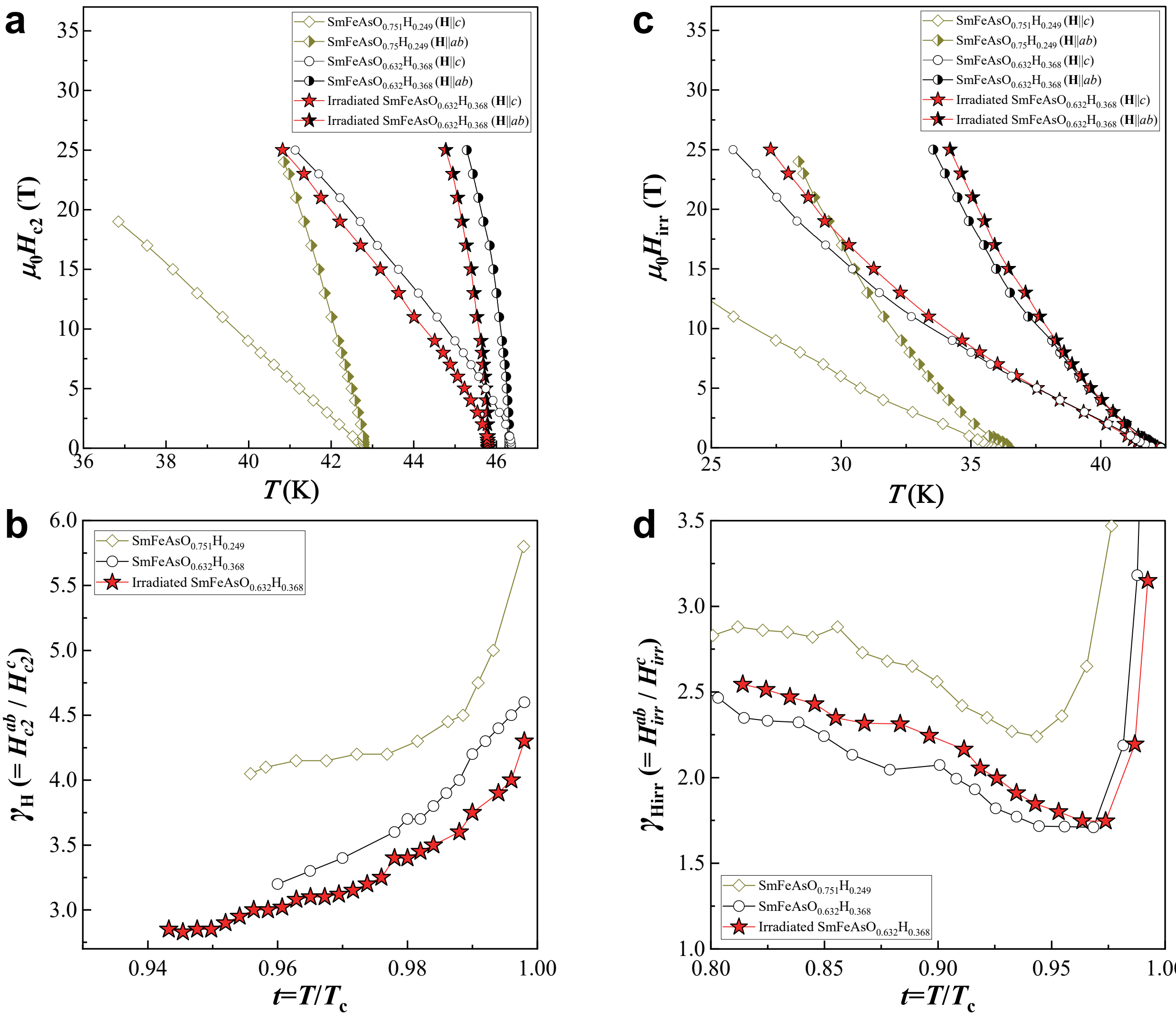

Figure S9: **(a)** Upper critical field (H_{c2}), **(b)** anisotropy of $H_{c2}(t)$, **(c)** irreversibility field (H_{irr}) and **(d)** anisotropy of $H_{irr}(t)$ for H-doped $SmFeAsO_{1-x}H_x$ films. Criteria of $0.8\rho_{normal}$ and $0.01\rho_{normal}$ are used to determine H_{c2} and H_{irr}., respectively.

(9) The parameters for the calculation of J_d at 4.2 K for different superconductors

Details of the calculation parameters for J_d at 4.2 K used in **Fig. 6b** are shown in **Table S4**.

Table S4: Parameters used to calculate J_d at 0 K and 4.2 K for various superconducting materials. The J_d(4.2K) is calculated used the following the Tinkham formula within Ginzburg-Landau theory $J_d(T) = \frac{\phi_0}{3\sqrt{3}\pi\mu_0\lambda_{ab}^2(T)\xi_{ab}(T)} \propto \left[1-\left(\frac{T}{T_c}\right)\right]^{3/2}$ [10].

Film material	T_c^{onset} (K)	$\xi_{ab}(0)$ (nm)	$\lambda_{ab}(0)$ (nm)	$J_d(0)$ (MA /cm^2)	J_d(4.2K) (MA /cm^2)	J_c(4.2K, Self-field) (MA /cm^2)	J_c(4.2K) / J_d(4.2K) (%)
$SmFeAsO_{1-x}H_x$ film							
x=0.249	43.93	1.81	150	247.5	212.9	4.11	1.9
x=0.339	46.51	1.52	135	363.9	315.7	7.12	2.3
x=0.368	46.55	1.46	129	414.9	360.0	9.22	2.6
Irradiated $SmFeAsO_{1-x}H_x$ film							
x=0.368	46.15	1.45	129	417.8	362.0	16.11	4.5
$YBa_2Cu_3O_y$ film							
p=0.142	87.3	2.37 [3]	145 [3]	202.3	187.9	19.20	10.2
p=0.160	92.2	1.95 [3]	129 [3]	310.6	289.7	27.00	9.3
p=0.177	89.8	1.66 [3]	114 [3]	467.3	434.9	38.68	8.9
$YBa_2Cu_3O_y$ +$BaHfO_3$ film							
p=0.177	89.8	1.66 [3]	114 [3]	467.3	434.9	91.23	21.0
$BaFe_2(As_{1-x}P_x)_2$ film							
x=0.33	28.1	2.29 [3]	216 [3]	94.3	74.0	3.55	4.8
x=0.45	21.5	3.46 [3]	264 [3]	41.8	30.2	1.35	4.5
x=0.50	18.0	4.50 [3]	258 [3]	33.7	22.6	0.96	4.2
x=0.60	9.8	5.90	302	18.7	8.1	0.45	5.6
$BaFe_2(As_{1-x}P_x)_2$ +$BaZrO_3$ film							
x=0.33	28.1	2.29 [3]	216 [3]	94.3	74.0	7.40	10.0
$FeSe_{1-x}Te_x$ film							
x=0.00	9.5	3.85 [15]	685 [15]	5.6	2.3	0.29	12.6
x=0.20	21.6	2.40 [15]	499 [15]	16.9	12.2	1.43	11.7
x=0.40	16.07	2.05 [15]	673 [15]	10.9	6.9	0.85	12.3
x=0.50	13.02	2.12 [15]	774 [15]	7.9	4.4	0.48	10.9

References

51. Gurevich, A. Upper critical field and the Fulde-Ferrel-Larkin-Ovchinnikov transition in multiband superconductors. *Phys. Rev. B* **82**, 184504 (2010).
52. Tarantini, C. et al., Significant enhancement of upper critical fields by doping and strain in iron-based superconductors. Phys. Rev. B **84**, 184522 (2011).
53. Watanabe, K., Yoshida, K., Aoki, T. & Kohjiro, S. Kinetic Inductance of Superconducting Coplanar Waveguides. Jpn. J. Appl. Phys. **33**, 5708 (1994).
